# *GRIN2B*-related neurodevelopmental disorder: current understanding of pathophysiological mechanisms

**DOI:** 10.3389/fnsyn.2022.1090865

**Published:** 2023-01-10

**Authors:** Shasta L. Sabo, Jessica M. Lahr, Madelyn Offer, Anika LA Weekes, Michael P. Sceniak

**Affiliations:** ^1^Department of Biology, Central Michigan University, Mount Pleasant, MI, United States; ^2^Program in Biochemistry, Cell and Molecular Biology, Central Michigan University, Mount Pleasant, MI, United States; ^3^Program in Neuroscience, Central Michigan University, Mount Pleasant, MI, United States

**Keywords:** GRIN2B, GluN2B (NMDA receptor subunit NR2B), dendrite development, autism (ASD), neuron development, disease variants, synapse development, NMDAR (NMDA receptor)

## Abstract

*The GRIN2B*-related neurodevelopmental disorder is a rare disease caused by mutations in the *GRIN2B* gene, which encodes the GluN2B subunit of NMDA receptors. Most individuals with *GRIN2B*-related neurodevelopmental disorder present with intellectual disability and developmental delay. Motor impairments, autism spectrum disorder, and epilepsy are also common. A large number of pathogenic *de novo* mutations have been identified in *GRIN2B*. However, it is not yet known how these variants lead to the clinical symptoms of the disease. Recent research has begun to address this issue. Here, we describe key experimental approaches that have been used to better understand the pathophysiology of this disease. We discuss the impact of several distinct pathogenic *GRIN2B* variants on NMDA receptor properties. We then critically review pivotal studies examining the synaptic and neurodevelopmental phenotypes observed when disease-associated GluN2B variants are expressed in neurons. These data provide compelling evidence that various GluN2B mutants interfere with neuronal differentiation, dendrite morphogenesis, synaptogenesis, and synaptic plasticity. Finally, we identify important open questions and considerations for future studies aimed at understanding this complex disease. Together, the existing data provide insight into the pathophysiological mechanisms that underlie *GRIN2B*-related neurodevelopmental disorder and emphasize the importance of comparing the effects of individual, disease-associated variants. Understanding the molecular, cellular and circuit phenotypes produced by a wide range of *GRIN2B* variants should lead to the identification of core neurodevelopmental phenotypes that characterize the disease and lead to its symptoms. This information could help guide the development and application of effective therapeutic strategies for treating individuals with *GRIN2B*-related neurodevelopmental disorder.

## 1 Introduction

*GRIN2B*-related neurodevelopmental disorder is a rare disease caused by mutations in the *GRIN2B* gene. In a large cohort of individuals diagnosed with neurodevelopmental disorders, the prevalence of *de novo GRIN2B* variants that are likely pathogenic was 0.2% (Platzer et al., 2017). *GRIN2B*-related neurodevelopmental disorder affects both males and females. The most common clinical phenotypes associated with disruptive *GRIN2B* variants are intellectual disability (ID) and developmental delay (DD; Platzer and Lemke, 2018; Hansen et al., 2021; ClinVar^1^). ID and DD range in severity, from mild to profound. Other common clinical phenotypes include muscle tone abnormalities or movement disorder, epilepsy, and autism spectrum disorder (ASD). Microcephaly, malformations of cortical development observed in MRI, and cortical visual impairment have also been documented. Up-to-date information on identified *GRIN2B* variants is compiled in several databases^2^^,^^3^^,^^4^^,^^5^^,^^6^. Information, such as patient symptoms and the current assessment of pathogenicity, can be found for individual variants^2^.

Several lines of evidence strongly suggest that deleterious *GRIN2B* variants are pathogenic. For rare diseases caused by *de novo* variants, the identification of multiple affected individuals with mutations within the same gene lends strong support for that gene being a *bona fide* causal gene. This is especially true if the gene is resistant to mutation, as evidenced by a low incidence of mutation within the healthy population. Mutations identified in *GRIN2B*-related neurodevelopmental disorder patients are *de novo* variants that are not found in siblings, parents, or other healthy individuals. Moreover, the *GRIN2B* gene is highly intolerant to mutation, bearing fewer variations in the healthy population than almost 99% of all other genes (Petrovski et al., 2013). Further supporting causality, hundreds of variants have been identified in patients with overlapping symptoms. For example, whole exome sequencing of sporadic ASD cohorts identified multiple *de novo* genetic variants that were strictly associated with ASD: these well-controlled studies and rigorous data analyses implicated *GRIN2B* as a high-confidence ASD gene (O’Roak et al., 2011; 2012; 2014; Sanders et al., 2012, 2015; de Rubeis et al., 2014; Iossifov et al., 2014; Stessman et al., 2017). Additional sequencing of *GRIN2B* in individuals with ID and other neurodevelopmental disorders has identified a large number of disease-associated *GRIN2B* variants (Endele et al., 2010; Myers et al., 2011; Tarabeux et al., 2011; Talkowski et al., 2012; Yoo et al., 2012; Kenny et al., 2014; Lemke et al., 2014; Pan et al., 2015; Swanger et al., 2016; Takasaki et al., 2016; Platzer et al., 2017).

According to the ClinVar database^1^, “pathogenic” or “likely pathogenic” variants in *GRIN2B* include single amino acid changes, truncations, and splice site mutations. Splice site variants are predicted to produce either truncation, exon skipping, or haploinsufficiency due to nonsense-mediated RNA decay. Numerous larger chromosomal abnormalities in the region of chromosome 12 that contains *GRIN2B* have also been reported. However, in these cases, it is not yet known which disease phenotypes are caused by *GRIN2B* vs. other genes affected by these large deletions and duplications.

## 2 Disease-Causing *GRIN2B* Variants Disrupt GluN2B, A Developmentally-Expressed Subunit of NMDA Receptors

The *GRIN2B* gene encodes GluN2B (NM_000834.4). GluN2B is a subunit of the NMDA receptor (NMDAR). NMDARs are ionotropic glutamate receptors that play a crucial role in glutamatergic synaptic transmission, synaptic plasticity, and development (Vieira et al., 2020; Hansen et al., 2021). NMDARs have intrinsic ion channels that are permeable to sodium, potassium, and calcium. NMDAR channels produce EPSCs in the postsynaptic cell that activate slower (several milliseconds) and persist for longer than other types of ionotropic glutamate receptors (Hansen et al., 2021). NMDARs are known as “coincidence detectors” since their ionotropic activity requires simultaneous ligand binding and depolarization-dependent removal of magnesium ions from the channel pore. In addition to contributing to synaptic EPSCs, NMDAR activation can produce long-lasting effects since calcium that enters through the NMDAR channel can bind to intracellular calcium-binding proteins and act as a second messenger to regulate a variety of downstream cellular processes, such as AMPA receptor trafficking and transcription (Hansen et al., 2021). Although NMDARs are most abundant in neurons, NMDARs have also been observed in astrocytes (although GluN2B does not appear to be as strongly expressed as other NMDAR subunits; Skowrońska et al., 2019) and activated microglia (Acarin et al., 1997; Murugan et al., 2011).

NMDARs are hetero-tetramers composed of two obligate GluN1 subunits combined with two GluN2 or GluN3 subunits. GluN2 subunits (GluN2A, GluN2B, GluN2C, and GluN2D) are encoded by four distinct genes (*GRIN2A*, *GRIN2B*, *GRIN2C*, and *GRIN2D*, respectively). NMDARs can be either di-heteromeric, containing two of the same GluN2 or GluN3 subunit, or tri-heteromeric, containing two different GluN2 and/or GluN3 subunits. NMDAR activation requires the binding of glutamate and a co-agonist, glycine or D-serine. The co-agonist is thought to be tonically bound to GluN1, although at sub-saturating levels (Hansen et al., 2021).

The subunit composition determines NMDAR properties. GluN2 subunits contain the glutamate binding site but GluN3 subunits do not; therefore, receptors must contain a GluN2 subunit to be capable of responding to glutamate (Wyllie et al., 2013; Myers et al., 2019). NMDAR time constants, open probabilities, and sensitivity to glutamate all depend on subunit composition. For example, the deactivation time course of GluN2B-containing receptors is more than five times longer than that of GluN2A-containing receptors. In addition, GluN2B-containing receptors desensitize more slowly than GluN2A-containing receptors (Wyllie et al., 2013; Myers et al., 2019).

GluN2B is 1,484 amino acids long and is comprised of several functional domains (Hansen et al., 2021). At the amino terminus lies the large extracellular amino-terminal domain (ATD). GluN2B also contains four membrane-bound domains (M1–M4). M1, M3, and M4 are transmembrane domains, while M2 is a re-entrant loop that does not traverse the membrane. The ligand-binding domain (LBD), responsible for glutamate binding, is comprised of two parts that are derived from sequences located prior to the first transmembrane domain (referred to as S1) and the extracellular loop located between the third and fourth membrane domains (referred to as S2). The carboxy terminal domain (CTD) comprises a large intracellular tail that contains protein-protein interaction sequences and sites for post-translational modifications, which regulate receptor trafficking and signaling (Vieira et al., 2020). Disease-associated variants have been identified in each functional domain of GluN2B (Hu et al., 2016).

**Figure 1:**
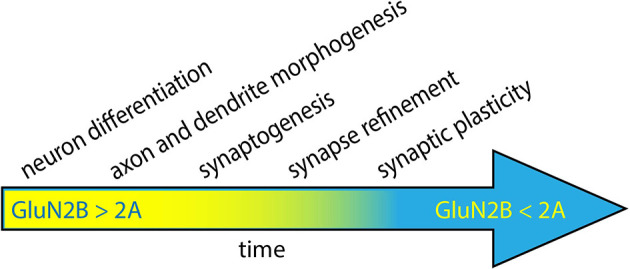
Neuronal development progresses through several steps, coinciding with the period of high expression of GluN2B. During the early stages of development, GluN2B levels progressively increase, while GluN2A levels are low (*yellow*). As neurons mature, GluN2A levels increase and ultimately surpass GluN2B expression (*blue*). GluN2B has been proposed to play a role in neuronal differentiation, dendrite morphogenesis, synaptogenesis, circuit refinement, and synaptic plasticity.

NMDARs containing GluN2A and/or GluN2B are most prevalent in glutamatergic synapses within the central nervous system; however, subunit composition varies with age, brain area, and cell type. GluN2B expression begins during embryonic development, with levels increasing as development continues (Figure 1; Ewald and Cline, 2008). During rodent postnatal development, most NMDARs in the cortex and hippocampus contain GluN2B, with levels peaking during the 3rd postnatal week (Watanabe et al., 1992; Komuro and Rakic, 1993; Monyer et al., 1994). Thus, GluN2B is the most abundant GluN2 subunit during both prenatal and postnatal development. GluN2A, on the other hand, becomes more common as neurons mature. In mature neurons, GluN2B expression persists, but GluN2A is more abundant in postsynaptic densities (PSDs) for many cell types (Figure 1; Vieira et al., 2020).

The transition from predominantly GluN2B expression to predominantly GluN2A is referred to as the 2A/2B switch. The timing of this switch is modulated by synaptic activity and generally coincides with developmental critical periods: windows of circuit development and refinement that are particularly sensitive to experience/activity (Myers et al., 2019). Since critical periods occur at different times in different brain regions, the timing of the 2A/2B switch varies by brain area. Strikingly, the induction of LTP in young rats leads to a rapid switch from GluN2B to GluN2A-containing receptors, which decreases the decay time of EPSCs generated in that same pathway (Bellone and Nicoll, 2007). These factors lead to a scenario where different PSDs of the same neuron may have different 2A/2B ratios, depending on presynaptic cell identity and history of synaptic plasticity.

The subcellular localization of GluN2B is also developmentally regulated. Early in neuronal development, GluN2B-containing NMDARs are observed throughout the dendrites. As synaptogenesis proceeds and neurons mature, GluN2B localization becomes increasingly punctate and concentrated at postsynaptic sites. While the studies of disease-associated GluN2B variants have focused on dendritic NMDARs, it is worth noting that GluN2B variants may also affect neurodevelopment through presynaptic mechanisms since GluN2B-containing receptors are found in a subset of axons and presynaptic terminals in developing cortex and hippocampus (DeBiasi et al., 1996; Charton et al., 1999; Jourdain et al., 2007; McGuinness et al., 2010; Larsen et al., 2011, 2014; Berg et al., 2013; Gill et al., 2015).

Given its spatial and temporal expression patterns, GluN2B has been hypothesized to play roles in a variety of neurodevelopmental processes, including neuronal differentiation, dendritogenesis, synaptogenesis, circuit formation/refinement, and synaptic plasticity (Figure 1; Ewald and Cline, 2008; Myers et al., 2019; Vieira et al., 2020). A large number of experiments have been performed to test each of these hypotheses, so it is beyond the scope of this review to discuss all of them here. However, seminal work has demonstrated that GluN2B is required for normal neural development since *Grin2b* knockout mice die perinatally (Kutsuwada et al., 1996). Remarkably, loss of GluN2B is not rescued by early expression of GluN2A *via* genetic replacement of GluN2B with GluN2A (Wang et al., 2011). Heterozygous *Grin2b* knockout mice survive, so one copy of wild-type *Grin2b* is sufficient for the essential functions of GluN2B.

### 2.1 Strategies for defining the pathophysiological mechanism of *GRIN2B*-related disorder

Given the complexity of NMDAR expression patterns, subcellular trafficking, and function, understanding the pathophysiology of *GRIN2B*-related neurodevelopmental disorder requires systematic investigation into the molecular and cellular phenotypes generated by disease-associated variants.

In general, studies of GluN2B variant pathophysiology can be categorized into three types of analysis: (1) Mutant GluN2B subunits are investigated *in silico* and in heterologous expression systems, with a focus on whether disease variants are efficiently delivered to the cell surface and how channel function is intrinsically affected; (2) GluN2B variant trafficking and channel function are studied in the context of wild-type GluN2 subunits, as would be expected in the disease state. Such experiments are performed in neurons, where wild-type GluN2 subunits are endogenously expressed, or in heterologous expression systems that co-express wild-type and mutant GluN2 subunits; and (3) the effects of disease-causing variants on neuron development, morphology, and physiology are examined, *in vitro* or *in vivo*. Below, we will discuss the progress that has been made at each of these levels of analysis. Since recent comprehensive reviews have provided excellent compilations of results obtained with heterologous expression systems, we will primarily focus on advances made in understanding the pathophysiological mechanisms of putative disease-causing *GRIN2B* variants in neurons.

### 2.2 Analysis of mutant subunit trafficking and currents in the absence of wild-type GluN2

The majority of studies of the impact of *GRIN2B* variants have utilized heterologous expression systems. Studies in heterologous cells provide the advantage of relatively quick and straight-forward analysis of the effects of each variant on NMDAR surface expression and channel properties. Expression of mutant GluN2B, along with GluN1, allows well-controlled, systematic analysis of variant function, without complications that may be introduced by co-expression with wild-type GluN2 subunits. This permits efficient assessment of whether each variant likely corresponds to a loss-of-function (LoF) or gain-of-function (GoF) variant and informs subsequent experimental designs. Tables containing much of these data are conveniently available on the CFERV website^7^, as well as in recent publications (Hu et al., 2016; Platzer et al., 2017; Myers et al., 2019; Amin et al., 2021; Kellner et al., 2021). Data for individual variants can be found at the GRINdb variant database, which curates data from a variety of sources (García-Recio et al., 2021).

Most NMDARs in the cortex and hippocampus contain GluN2B, with levels peaking during the third postnatal week. A variety of strategies have been used to examine whether mutant GluN2B is properly trafficked, including surface immunolabeling of unpermeabilized (or mildly permeabilized) cells (Liu et al., 2017; Sceniak et al., 2019; Santos-Gómez et al., 2021), surface biotinylation-based approaches (Ogden et al., 2017), and fluorescent/luminescent/colorimetric reporter methods (Swanger et al., 2016; Amin et al., 2018; Li et al., 2019; Kellner et al., 2021). These studies showed that some variants produce GluN2B that is not trafficked to the cell surface, while others are trafficked less efficiently than or equivalent to wild-type.

Receptors that are never delivered to the cell surface cannot function as plasma membrane receptors and ion channels; for receptors that reach the cell surface, it is necessary to measure their channel properties directly. Electrophysiology and calcium imaging have been used to evaluate channel function (Swanger et al., 2016; Ogden et al., 2017; Amin et al., 2018; Fedele et al., 2018; Vyklicky et al., 2018; Li et al., 2019; Sceniak et al., 2019; Shin et al., 2020; Kellner et al., 2021; Santos-Gómez et al., 2021; Elmasri et al., 2022). Some mutants form NMDARs possessing channels that are completely non-functional. Other mutant variants have abnormal functional properties, including abnormal glutamate potency, altered desensitization, or changes in the ability of magnesium to block currents. Despite the significant effort toward uncovering the properties of GluN2B mutants, only a relatively small fraction of the identified *GRIN2B* variants have been evaluated (estimated at <15%; Kellner et al., 2021). Therefore, much work remains to be completed in order to develop a full understanding of known GluN2B variants.

### 2.3 Analysis of mutant subunit trafficking and currents in the presence of wild-type GluN2

Afflicted individuals are heterozygous for *GRIN2B* variants. This raises some important questions:

• Do disease-associated GluN2B variants co-assemble with wild-type GluN2B subunits into hybrid mut/wt NMDARs?
• Does mutant GluN2B form tri-heteromeric receptors with GluN2A?
• How does the incorporation of mutant subunits into receptors containing wild-type 2A or 2B affect receptor function?
• Do mutant subunits act in a dominant-negative manner to override wild-type subunits within hybrid receptors? For example, if mutant GluN2 subunits are capable of co-assembling into receptors with wild-type GluN2 subunits, mutant subunits could interfere with trafficking or activity of co-assembled wild-type subunits.
• If glutamate-binding is required for NMDAR trafficking to the plasma membrane, do LBD mutant subunits sequester wild-type subunits in the biosynthetic pathway?
• Does the presence of disease variants alter the expression of wild-type GluN2B or GluN2A at synapses or the timing of the 2A/2B switch?
• Which GluN2B variants can be adequately modeled as simple haploinsufficiency?

Recent experiments have begun to address these questions by examining mutant localization and function in the presence of wild-type subunits. Several studies have examined the effects of disease-associated GluN2B variants in heterologous systems that co-express wild-type and mutant GluN2B (Swanger et al., 2016; Amin et al., 2018; Li et al., 2019; Kellner et al., 2021; Elmasri et al., 2022). Together, these studies suggest that GluN2B variants are capable of co-assembly with wild-type GluN2B subunits.

While heterologous expression in nonneuronal cells produces valuable insights, it is important to further evaluate variants in the context of neurons for several reasons. Since neurons are highly compartmentalized, regulation of NMDAR trafficking in neurons involves not only forward trafficking through the biosynthetic pathway and to the cell surface but also appropriate sorting into axons and dendrites. Whether NMDARs are sorted into axons varies with neuron age and subtype (Fedder and Sabo, 2015). Furthermore, dendritic NMDARs are differentially localized (e.g., to postsynaptic densities, extrasynaptic sites, dendrite shafts, or intracellular pools), depending on subunit composition, neuron subtype, neuronal activity, and age (Vieira et al., 2020). In addition, neuronal NMDAR expression, trafficking, and function are regulated by a variety of posttranslational modifications, signal transduction pathways, and protein-protein interactions, which may be absent in heterologous systems (e.g., Sans et al., 2003; Chung et al., 2004; Prybylowski et al., 2005; Hayashi et al., 2009). Thus, dysregulation of NMDAR expression, trafficking, and function by such pathways may be integral to the pathophysiological mechanisms of *GRIN2B*-related neurodevelopmental disorder.

### 2.4 Localization of GluN2B disease variants in neurons

It has been shown that ligand-binding is necessary for proper trafficking of NMDARs (She et al., 2012), and LBD variants tend to affect glutamate binding and gating (Amin et al., 2021). This raises the question of whether variants in the LBD—or that otherwise interfere with ligand binding—prevent trafficking of mutant receptors to postsynaptic densities. To test this, two variants in S1 of the LBD (E413G and C461F) were studied in neurons, cultured at E18 for 12 DIV (Swanger et al., 2016). Both variants were identified in individuals with ID. As predicted, both variants were less efficiently delivered to dendrites, and the fraction of GluN2B-E413G and C461F localized to the surface of dendrites was reduced compared to wild-type GluN2B (Swanger et al., 2016). These results were consistent with reduced surface expression of E413G and C461F observed in a non-neuronal expression system (Swanger et al., 2016). It is worth noting that a recently described variant, G689S, exhibited an EC50 for glutamate that is orders of magnitude lower than wild-type GluN2B, but this mutant was trafficked to the plasma membrane in *Xenopus* oocytes (Kellner et al., 2021). This result appears to conflict with the conventional idea of a link between ligand binding and forward trafficking, but the surface expression of this variant was not directly evaluated in neurons.

Numerous variants have been identified that are predicted to truncate GluN2B within S2 of the LBD (Endele et al., 2010; O’Roak et al., 2011; Lemke et al., 2014; Kenny et al., 2014; Platzer et al., 2017; Stessman et al., 2017). One of these, a single-base substitution (c27172-2A>G) at the canonical 3’ splice site of exon 10 in the GluN2B subunit, has been studied in neurons (Sceniak et al., 2019; Bahry et al., 2021). This variant was identified in a female with severe nonsyndromic ASD and ID (O’Roak et al., 2011) and has been modeled as a truncation at amino acid 724, deleting the cytoplasmic tail of GluN2B, the fourth transmembrane domain, and a substantial portion of the S2 extracellular loop. This mutant was not trafficked to the plasma membrane or into dendrites of rat cortical neurons (Sceniak et al., 2019). Mutant subunits were capable of interacting with wild-type receptor subunits, and homozygous mutant receptors completely lack calcium currents. Interestingly, restoration of TMD4 and the CTD did not rescue trafficking or function, suggesting that loss of S2 was sufficient to interfere with receptor trafficking.

Subsequently, additional truncated GluN2B proteins have also been examined: truncation at E839 (in TMD4) and R847 (proximal part of CTD; Santos-Gómez et al., 2021). The E839 variant was identified in a female reported as having clinical symptoms of ID, along with deficits in fine motor skills and abnormal EEG. The R847 variant was identified in a female with ID. Consistent with prior observations for truncation within S2 (Sceniak et al., 2019) and previous studies of the role of the CTD in surface delivery (Sans et al., 2003; Chung et al., 2004; Prybylowski et al., 2005), both E839 and R847 showed reduced surface expression when exogenously expressed in mouse hippocampal neurons. It is worth noting that, based on results with the R847 mutant, the CTD was not required for surface expression in transfected COS-7 cells. This discrepancy between trafficking to the cell surface in neurons and COS-7 cells underscores the importance of studying GluN2B variants in neurons.

### 2.5 Effects of GluN2B disease-associated variants on NMDAR currents

To understand the role of channel function in disease pathophysiology, it is important to characterize synaptic NMDAR responses in the presence and absence of GluN2B disease variants. To study the electrophysiological responses of NMDARs containing mutant GluN2B, multiple experimental paradigms have been applied. Since the relative contributions of GluN2B and other GluN2 subunits vary with age and cell type, these parameters need to be considered while interpreting and comparing the results of the experiments outlined below. For example, in the cortex and hippocampus, tri-heteromeric receptors in which mutant GluN2B co-assembles with GluN2A are more likely to be observed after the 2A/2B switch than earlier in development.

One experimental model is to use molecular replacement. Endogenous, wild-type receptors are eliminated, typically through Cre-dependent knockout in GluN2B^flox/flox^ mice or GluN2A^flox/flox^ + GluN2B^flox/flox^ mice. Then, either mutant or wild-type GluN2B is expressed on the knockout background. When both GluN2A and Glu2B are removed, mutant receptors can be studied in the absence of wild-type GluN2 subunits. When only GluN2B is eliminated, endogenous GluN2A remains available to co-assemble with mutant subunits into tri-heteromeric receptors.

In one example of this type of experiment, Fedele et al. (2018) examined the effects of a series of GluN2B variants on evoked EPSCs in hippocampal neurons, cultured at E18, transfected at 10 DIV and analyzed at 13–15 DIV. Four disease-associated variants were examined: C461F in S1 (associated with Lennox Gastaut syndrome and ASD), P553L in preM1 (associated with ID), and two variants in the M2-M3 linker, N615I and V618G (associated with West Syndrome). 3D models of NMDAR structure were used to guide the selection of these mutants. All but V618G exhibited accelerated decay kinetics, while only P553L reduced EPSC amplitude (Fedele et al., 2018). Interestingly, N615I and V618G mutant receptors were significantly less sensitive to magnesium block, leading to the hypothesis that these mutants act as gain-of-function variants (Fedele et al., 2018).

Another useful approach is to exogenously express mutant (or wild-type) GluN2B on a complete wild-type background. Here, mutant subunits are analyzed in the presence of wild-type GluN2B, so NMDARs may contain only mutant GluN2B, only wild-type GluN2 subunits, or mutant GluN2B co-assembled with wild-type GluN2. With this approach, it is ideal to determine whether transfection with GluN2B constructs produces over-expression of GluN2B. In most instances where this is reported, it appears as though levels of GluN2B in dendrites and synapses do not significantly increase from transfection with GluN2B-expressing cDNA (Fedele et al., 2018; Sceniak et al., 2019), suggesting that expression of endogenous, wild-type GluN2B is reduced to compensate for exogenous expression of GluN2B. When levels of GluN2B are not increased, this approach reasonably reflects the scenario in patients, who are heterozygous for these variants.

A final approach that has been employed is to create neurons or animals that are heterozygous for the mutation. This is generally accomplished by either using molecular replacement in heterozygous GluN2B knockouts or through the generation of germline knock-in animals. This experimental design closely reflects the disease state, with regard to heterozygosity for mutant GluN2B. As with the previous approach, wild-type GluN2 subunits are available for co-assembly with mutant GluN2B.

In a recent report from Elmasri et al. (2022), molecular replacement was used to compare the consequences of putative GoF (R696H, R540H) and LoF (C456Y and C461F) *GRIN2B* variants on synaptic function in neurons that lacked either GluN2B or both GluN2B and GluN2A. Cultured mouse hippocampal slices were made from P6–8 mice with floxed alleles of *Grin2a* + *Grin2b* or only *Grin2b*. A small number of CA1 neurons in the slices were transfected with Cre recombinase, along with various GluN2B variants, by single-cell electroporation at 6–8 DIV. Evoked SC-CA1 NMDAR-EPSCs (the NMDAR-mediated component of EPSCs) were recorded in transfected and untransfected cells in the same slices at 15–20 DIV. GoF mutants showed functional incorporation into CA1 synapses since mutant NMDAR-EPSCs were observed when both GluN2B and GluN2A were knocked out (Elmasri et al., 2022). Interestingly, the NMDAR-EPSCs recorded upon expression of disease-associated variants were dramatically different when GluN2A was available to compensate for mutant GluN2B. When the expression of endogenous GluN2A and GluN2B were knocked out, NMDAR-EPSCs were prolonged. However, in the presence of native GluN2A (but knock-out of endogenous GluN2B), disease-associated mutants produced no changes in NMDAR-EPSC peak amplitudes, while NMDAR-EPSC charge transfer was reduced to nearly half in LoF mutants and modestly reduced in GoF mutants. Through comparison with responses recorded in the absence of GluN2A and wild-type GluN2B and recordings from tri-heteromeric NMDARs in heterologous cells, it was concluded that mutant GluN2B subunits likely combine with wild-type GluN2A. It has previously been shown that GluN2A dominates the functional properties of tri-heteromeric GluN1/2A/2B receptors (Hansen et al., 2021). Effects of mutant GluN2B on postsynaptic function were cell-autonomous, given the sparse transfection used for these experiments. One important question that remains is whether these same mutants would have co-assembled with wild-type GluN2B (rather than or in addition to GluN2A) if the experiment had been performed in mice that were heterozygous for the floxed *Grin2b* allele. In the future, it would be interesting to know whether mutants that are inefficiently trafficked to dendrites or the cell surface are similarly incorporated into tri-heteromeric receptors in neurons.

### 2.6 Effects of mutant subunits on neuron development, morphology, and physiology

To understand how disease-linked variants lead to clinical phenotypes observed in *GRIN2B*-related neurodevelopmental disorder, it is necessary to understand how individual variants affect neuron and circuit development, neuronal morphology, and synaptic physiology and plasticity. NMDAR biology is complex, and so is nervous system development; therefore, it is difficult to predict the cellular and circuit-level outcomes of individual disease variants. Consequently, empirical evidence regarding cellular and circuit phenotypes of each type of mutation is needed to help guide future therapeutic development and effective delivery of personalized medicine.

*GRIN2B*-related neurodevelopmental disorder is typically discovered in pediatric patients, with the onset of symptoms often occurring during the first few years of age. The age of symptom onset and the prevalence of GluN2B during embryonic and early postnatal development suggest that stages of development that occur during this timeframe are likely to underlie disease pathogenesis (Figure 1). Below, we will describe key studies that explore the impact of GluN2B variants on embryonic and early postnatal development, including neuron differentiation (Bell et al., 2018), dendritogenesis (Sceniak et al., 2019; Bahry et al., 2021), synaptogenesis (Liu et al., 2017; Sceniak et al., 2019), and synaptic plasticity (Shin et al., 2020; Kellner et al., 2021).

#### 2.6.1 GluN2B variants interfere with neuronal differentiation

NMDARs appear to play a role in neural progenitor cell migration and differentiation (Balázs et al., 1988; Blanton et al., 1990; Brenneman et al., 1990; Behar et al., 1999; Cohen and Greenberg, 2008). GluN2B is the predominant GluN2 subunit during embryonic ages, suggesting that these functions are mediated by GluN2B-containing receptors. Given this information and the observation that around 14% of patients with *GRIN2B*-related neurodevelopmental disorders display cortical malformations in MRI (Platzer et al., 2017), it was hypothesized that pathogenic variants in GluN2B might act by interfering with neuronal differentiation (Bell et al., 2018).

**Figure 2:**
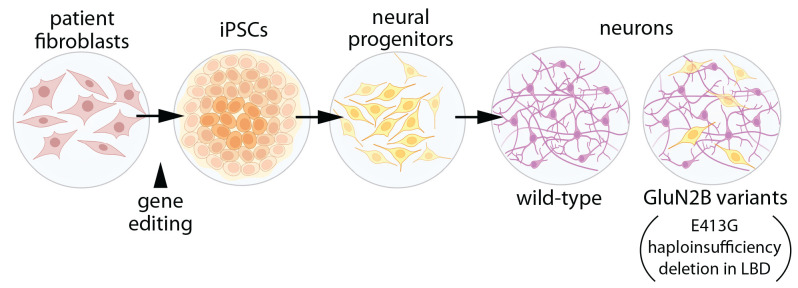
Expression of GluN2B variants interferes with neuronal differentiation. Induced pluripotent stem cells (iPSCs) were generated with either: (1) GluN2B harboring the disease-associated missense mutation, E413G, within the ligand-binding domain (LBD); (2) deletion of a section of the GluN2B LBD, or (3) GluN2B haploinsufficiency (Bell et al., 2018). Neural progenitor cells were generated from iPSCs, then differentiated into forebrain neurons. In differentiated cells with GluN2B variants, expression of genes related to cell proliferation and pluripotency was elevated, while genes associated with neuronal differentiation were reduced, when compared to cells with two alleles of wild-type GluN2B. Illustrations were partially created with BioRender.com.

To test this hypothesis, induced pluripotent stem cells were used to generate human forebrain neural progenitor cells (NPCs), which were then differentiated into human neurons (Figure 2; Bell et al., 2018). Three types of NPCs were evaluated to model *GRIN2B* disease variants: a frameshift mutant that eliminated mRNA from one allele of *GRIN2B* (essentially haploinsufficient), large deletions in the GluN2B LBD, and E413G (in the LBD). For E413G, the NPCs were derived from a patient presenting with ASD and mild ID (Bell et al., 2018). For all three types of NPCs, differentiation into neurons was suppressed. For E413G, the differentiation phenotype was rescued when the mutation was corrected. The effects of GluN2B appear to depend on NMDAR activation since they could be mimicked with APV and ifenprodil (inhibitors of NMDARs and GluN2B-containing NMDARs, respectively), and all mutants interfered with calcium influx through NMDARs. Given this reduced rate of differentiation, it would be interesting to know whether a slower rate of differentiation is sufficient to produce cortical malformations similar to those observed in patients. The observation that the competitive antagonist, APV, recapitulates the mutants’ effects on differentiation suggests that any variant that prevents ligand binding or produces haploinsufficiency might also impede neuron differentiation. If so, one might expect the prevalence of cortical malformations or microcephaly to be higher in the *GRIN2B* patient population, although more subtle defects in cortical patterning might evade detection by MRI.

#### 2.6.2 A GluN2B disease variant restricts dendritogenesis and spine maturation

Neural circuit connectivity and synaptic integration are shaped by dendrite architecture. Abundant evidence indicates that GluN2B influences dendrite growth and arborization (Ewald et al., 2008; Espinosa et al., 2009; Sepulveda et al., 2010; Bustos et al., 2014; Keith et al., 2019). Direct manipulation of GluN2B expression or genetic swapping of GluN2A with GluN2B is sufficient to induce abnormal dendrite architecture in a variety of neuron types (Ewald et al., 2008; Espinosa et al., 2009; Sepulveda et al., 2010; Bustos et al., 2014; Keith et al., 2019). Moreover, when GluN2B homozygous knockout neurons were compared to GluN2B heterozygous neurons *in vivo*, patterning of dendrites was abnormal in homozygous knockout neurons, although dendrite length and branching were unaltered in granule cells and cortical layer 4 spiny stellate cells in barrel cortex (Espinosa et al., 2009).

**Figure 3:**
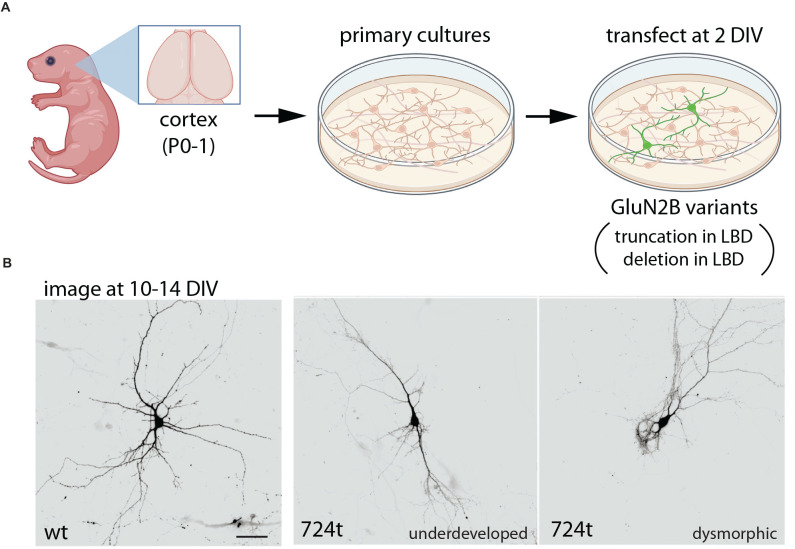
Expression of GluN2B variants interferes with dendrite morphogenesis. **(A)** Primary neuronal cultures were prepared from wild-type rat cortex, at postnatal day 0–1 (P0–1), then transfected at 2 days *in vitro* (DIV) with either wild-type GluN2B or GluN2B bearing a truncation at amino acid 724 (724t), within the LBD (Sceniak et al., 2019; Bahry et al., 2021). In parallel, GluN2B with a large depletion in the LBD was also examined, producing similar results. **(B)** In contrast to neurons expressing only wild-type GluN2B, neurons expressing mutant GluN2B had impaired dendrite development, with some neurons appearing under-developed and others appearing dysmorphic (displaying unusual dendritic structures). Overall, GluN2B variants produced fewer branches and reduced branch length. Illustrations were partially created with BioRender.com. Images in **(B)** are from Sceniak et al. (2019).

The impact of disease-associated GluN2B variants on dendrite morphogenesis was examined using a GluN2B variant that is associated with severe ASD and ID and has been predicted to truncate GluN2B at amino acid 724 (O’Roak et al., 2011; Sceniak et al., 2019; Bahry et al., 2021). As discussed above, NMDARs containing this truncation (724t) were non-functional. Expression of this variant in young cortical neurons led to dramatic defects in dendrite morphogenesis (Figure 3; culture at P0–P2, transfect at 2–3 DIV; Sceniak et al., 2019; Bahry et al., 2021). Dendrite maldevelopment occurred when mutant GluN2B was expressed in wild-type neurons, suggesting a dominant-negative mechanism. Mutant GluN2B was sparsely expressed in neurons; therefore, the observed effects on dendrite growth were cell-autonomous. As described above for trafficking of this mutant, deletion of a portion of the S2 extracellular loop was sufficient to reproduce the aberrant dendrite morphogenesis observed with the truncated GluN2B, raising the question of whether any variant that eliminates ligand-binding might produce similar dendrite morphology phenotypes (Sceniak et al., 2019).

Expression of GluN2B truncated in S2 (724t) or bearing a large deletion in S2 resulted in reduced dendrite length, complexity, and branching patterns (Sceniak et al., 2019; Bahry et al., 2021). Dendrites appeared underdeveloped in some neurons, while dendrites of other neurons were dysmorphic, appearing grossly abnormal. Overall, the total dendrite arbor length was dramatically reduced in neurons expressing mutant GluN2B as compared to neurons expressing wild-type GluN2B (Sceniak et al., 2019). Reduced dendrite length was evident in primary, intermediate, and terminal dendrite segments. Neurons expressing mutant GluN2B also displayed fewer branch points and fewer intermediate and terminal branches, whereas primary branching was similar in neurons expressing wild-type and mutant GluN2B (Sceniak et al., 2019).

In cortical pyramidal neurons, apical and basal dendrites are distinct subcellular compartments, and expression of mutant GluN2B leads to a net loss of terminal dendrites, but apical/principal dendrite branches were unaffected (Bahry et al., 2021). This observation suggests that such subcellular distinctions are important to consider in future studies of *GRIN2B* variant pathophysiology.

Dendritic arbors are established through the formation of new branches followed by branch elongation and subsequent stabilization or elimination (Lohmann and Wong, 2005; Yoong et al., 2019). In live imaging experiments, GluN2B truncated at amino acid 724 impeded dendrite development by reducing elongation and promoting dendritic pruning (Bahry et al., 2021). These data suggest that this class of GluN2B variants contribute to ASD and ID pathophysiology by shifting the dynamics of dendrite growth away from extension and toward branch elimination, thereby reducing dendritic arbor size and complexity and disrupting normal circuit development and function. Dendrite morphology directly affects the extent of synaptic connectivity, the number of potential synaptic partners a neuron interacts with, and dendritic filtering of postsynaptic responses (Ledda and Paratcha, 2017; Martínez-Cerdeño, 2017). This raises the question of whether the observed dendrite morphology defects lead to altered circuit composition, synaptic integration, and plasticity.

#### 2.6.3 Variable effects of GluN2B variants on synapse number and density

During synaptogenesis, competing processes of synapse formation and elimination determine the final number and density of synapses. Recent reports have begun to reveal how GluN2B variants affect synapse number and density. For example, dendritic spine density was unchanged in the presence of GluN2B truncated at amino acid 724 or with deletion of a portion of S2 in the LBD, while dendritic arbors were smaller (Sceniak et al., 2019), suggesting that total synapse number was reduced. In addition, dendritic spines appeared less mature in dendrites expressing mutant GluN2B when compared to spines of dendrites expressing only wild-type GluN2B (Sceniak et al., 2019).

In this study, dendritic spines were used as a proxy for synapses. It is worth noting that during normal postsynaptic development, immature synapses are often formed on dendrite shafts, rather than dendritic spines, then spines emerge as neurons mature. Given the predominant expression of GluN2B during early postnatal development, it will be interesting to use a synaptic marker that allows analysis of immature postsynaptic structures to determine whether GluN2B variants differentially affect shaft and spine synapses.

Liu et al. (2017) studied a variant that was identified in a patient with ASD, although it has not been shown that this variant is pathogenic (GRINdb^2^). Dissociated hippocampal neurons transfected with S1415L (S1413L in rodents) showed a modest 20% decrease in dendritic spine density compared to wild-type GluN2B (cultured at E18, transfected at 14 DIV and analyzed at 17 DIV; Liu et al., 2017), likely due to reduced trafficking to the postsynaptic plasma membrane. Interestingly, a similar reduction in spine density was observed with the deletion of the entire CTD (Liu et al., 2017). Given the predominant expression of GluN2B during early postnatal development, it would be informative to know how synaptogenesis would be affected if mutant GluN2B subunits were expressed earlier in development. It will also be interesting to determine whether similar changes in spine density are produced by additional variants that are clearly pathogenic and produce reduced localization at the postsynaptic plasma membrane.

**Figure 4:**
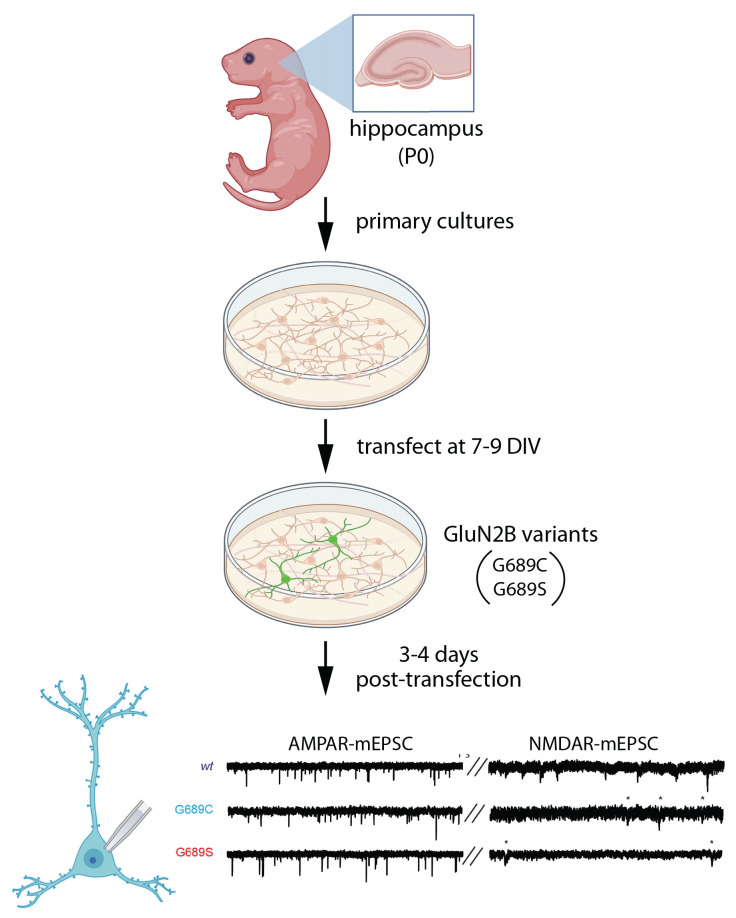
Expression of GluN2B variants does not affect synapse number but reduces NMDARs at synapses. Primary neuronal cultures were generated from P0 rat hippocampus, then transfected at 7–9 DIV with disease-associated GluN2B variants, G689C or G689S, in the LBD (Kellner et al., 2021). After 3–4 days, miniature excitatory postsynaptic currents (mEPSCs) were subjected to patch clamp recording, separating NMDAR- and AMPAR-mediated components of mEPSCs. AMPAR-mEPSC frequency was unchanged, suggesting no change in the number of synapses. However, the frequency of NMDAR-mEPSCs was reduced, likely due to an increase in synapses that lack NMDARs. Illustrations were partially created with BioRender.com. Recordings are from Kellner et al. (2021).

Recently, Kellner et al. (2021) examined the effects of two different variants in the same residue within the LBD, G689C and G689S, on spontaneous synaptic transmission (Figure 4). Both individuals experienced severe ID and DD, along with movement abnormalities. The G689C patient also had a cortical malformation in MRI and visual impairment (Kellner et al., 2021). When these variants were expressed in dissociated rat hippocampal cultures (plated at P0, transfected at 7–9 DIV, and recorded 3–4 days post-transfection), NMDA-mEPSC frequency was reduced dramatically in the presence of G689C and G689S (Kellner et al., 2021). Traditionally, a change in mini frequency is interpreted as either a presynaptic change or a change in synapse number. However, AMPAR-mEPSC frequency was unaffected by these variants. Assuming that nearly all synapses contain AMPARs that would be activated by spontaneous glutamate release, this implies that synapse number and probability of release are unchanged by the variants. Since these experiments were performed in wild-type neurons, which express wild-type GluN2B, the dramatic effect on synaptic NMDAR-mEPSCs suggests a dominant-negative effect of these disease-associated GluN2B variants. Consistent with this mechanism, in Xenopus oocytes, mutant subunits were able to co-assemble with wild-type GluN2B and exhibit dominant-negative effects (Kellner et al., 2021).

Two plausible explanations for the reduced frequency of mEPSCs are the presence of synapses that lack functional NMDARs or a reduction in the amplitudes of NMDAR-mEPSCs, rendering more EPSCs undetectable. When NMDAR-mEPSC amplitudes were quantified, G689 reduced mEPSC amplitude, whereas G689C modestly increased mEPSC amplitude. Both variants produced faster deactivation kinetics, as compared to wild-type NMDAR-mEPSCs, which may be due to a compensatory increase in GluN2A-containing receptors. These observations suggest that these mutants lead to the formation of synapses that lack functional NMDARs. Interestingly, in heterologous experiments in Xenopus oocytes, both G689C and G689S variants dramatically reduced the potency of glutamate, with 1,000–2,000-fold lower EC50 for glutamate. Therefore, synapses without functional NMDARs may be mainly populated with di-heteromeric receptors containing mutant GluN2B, which are insensitive to synaptic glutamate. It is worth noting that G689C was inefficiently transported to the plasma membrane in a heterologous expression system, while trafficking of G689S appeared normal (Kellner et al., 2021). Thus, both mechanisms are possible.

Together, the current data suggest that some variants likely perturb aspects of synaptogenesis, while others may have little effect on synapse formation and elimination. For variants that alter synapse density or number, it will be important to determine whether observed changes in synapse number/density are a result of defects in synapse formation and/or elimination. To distinguish between these possibilities, it will be necessary to perform live-imaging to observe each process directly. It would also be useful to examine how the early expression of GluN2B variants affects these same processes since synapse formation begins prior to the expression window for the experiments described above with S1415/1413L and G689C/S. Given the NMDAR half-life, earlier expression of mutant GluN2B could increase the percentage of NMDARs that contain mutant receptors during synaptogenesis and produce more pronounced effects on synapse density or maturation.

#### 2.6.4 GluN2B variants limit synaptic plasticity

**Figure 5:**
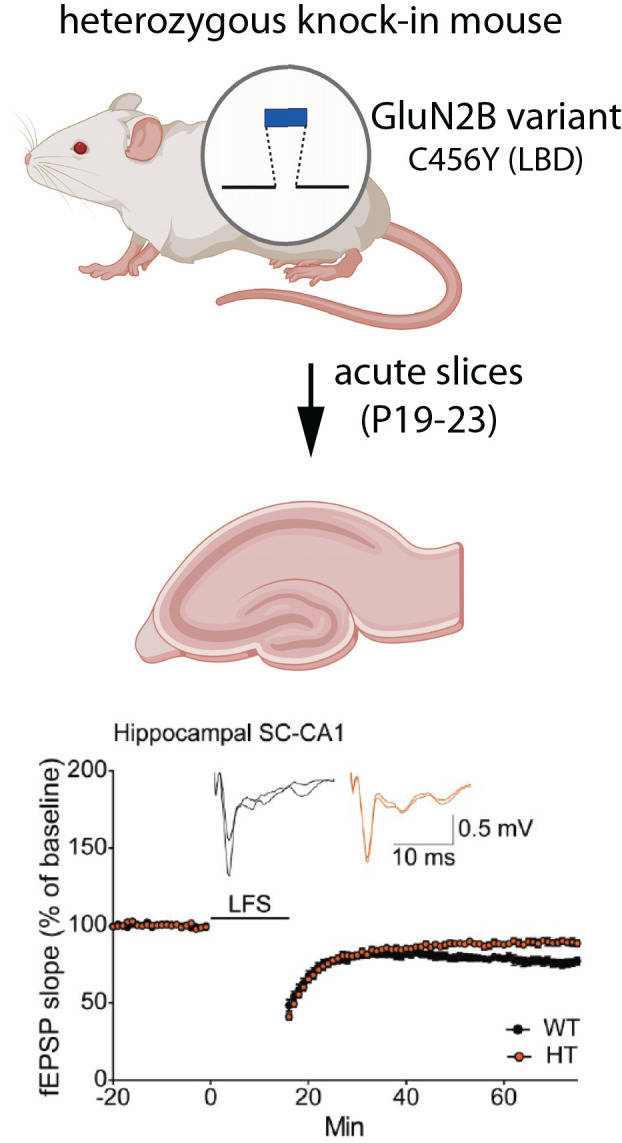
Expression of a GluN2B variant reduces LTD. Mice were generated that were heterozygous for the disease-associated missense variant, C456Y, in the LBD (Shin et al., 2020). At postnatal day 19–23, field EPSPs (fEPSPs) were recorded from CA1 neurons in acute hippocampal slices. Neurons expressing the C456Y variant had impaired NMDAR-dependent LTD, induced by low frequency stimulation (LFS). Synapse density and postsynaptic ultrastructure were unaffected. Illustrations were partially created with BioRender.com. Recordings are from Shin et al. (2020).

The role of GluN2B in synaptic plasticity is well-established. To examine the effects of *GRIN2B* disease variants on synaptic plasticity, Shin et al. (2020) generated a knock-in mouse line to replace wild-type GluN2B with mutant GluN2B (C456Y, located within the LBD) *in vivo* (Figure 5). This variant was initially identified as a *de novo* mutation in a male with ASD and ID (O’Roak et al., 2012). Importantly, comparisons were made between wild-type animals and those that were heterozygous for the C456Y variant, closely reflecting the human *GRIN2B* genetic state. Knock-in of mutant GluN2B reduced the GluN2B-mediated component of EPSCs in hippocampal SC-CA1 synapses, as assessed at P19–23. NMDAR-dependent long term depression (LTD) was also significantly impaired at these synapses. Interestingly, an even stronger loss of NMDAR-dependent LTD was observed at synapses in layer 1 of mPFC, underscoring the importance of considering brain area and cell type. Surprisingly, C456Y mutant GluN2B did not affect the density or ultrastructure of PSDs in CA1. The mEPSC frequency and amplitudes were also unchanged, consistent with no difference in synapse number or AMPA receptor mediated responses. These observed electrophysiological changes were mostly phenocopied by conventional heterozygous knockout of GluN2B in parallel experiments and previous reports (Kutsuwada et al., 1996).

A key question is whether the expression of mutant GluN2B results in compensatory changes in the expression of other GluN subunits. Although the C456Y mutant protein was expressed at lower levels than wild-type GluN2B, in both whole brain homogenates and synaptosomes, GluN2A was not upregulated to compensate for the reduction in GluN2B (Shin et al., 2020). Interestingly, at postnatal ages when NMDA receptors in the hippocampus and cortex predominantly contain GluN2B (rather than GluN2A), a concomitant reduction in GluN1 was observed. In contrast, at older ages when GluN2A typically replaces GluN2B at many synaptic NMDARs, GluN1 was unchanged, despite a continued reduction in GluN2B (Shin et al., 2020). In the future, it will be important to determine whether the expression of wild-type subunits is affected by additional GluN2B variants. Furthermore, given that the 2A/2B switch is occurring during the time window examined in C456Y knock-in mice, it remains possible that compensatory changes in subunit expression might still occur at younger ages, when synapses contain predominantly GluN2B, possibly accelerating or delaying the timing of the switch.

## 3 Characterization of *GRIN2B* Variant Pathophysiology Lays The Groundwork for Personalized Medicine

Recent studies have explored potential treatment options in patients with *GRIN*-related disorders by considering the impact of specific *GRIN* variants on NMDAR function. Such studies suggest that treatments can be guided by the classification of a variant as either GoF or LoF. In several patient studies, molecular modeling of *GRIN* variants was used to predict the functional properties the variant imposes on NMDARs in order to create a personalized treatment plan (Pierson et al., 2014; Soto et al., 2019; Xu et al., 2021; Krey et al., 2022). For patients with GoF *GRIN* variants, one intriguing possibility is using the NMDAR antagonist, memantine, to dampen excess activity. One study reported on the experimental treatment of a pediatric patient with a GoF *GRIN2A* variant, characterized by epileptic encephalopathy and severe cognitive impairment. After 2 weeks of daily treatment with memantine (titrated up to ~0.5 mg/kg), the frequency of seizures decreased considerably. A decrease in seizures was seen for the duration of the recorded period of over a year (Pierson et al., 2014). Another study followed daily treatment with memantine (~0.4 mg/kg) in a pediatric patient with a GoF *GRIN1* variant. This patient also presented with epileptic encephalopathy and severe developmental delay. Following 3-month memantine treatment, the patient’s weekly seizure rate was reduced dramatically and improvements in behavior were seen (Xu et al., 2021). For the treatment of patients with LoF *GRIN* variants, studies have assessed the effects of promoting NMDAR activity with L-serine, a precursor to the co-agonist, D-serine. One study reported L-serine treatment in a pediatric patient with an LoF *GRIN2B* variant. Following daily L-serine supplementation (~500 mg/kg) for 17 months, improvements were seen in motor, behavioral and social function, including the new-found ability to communicate with gestures (Soto et al., 2019). Another article reported that after 2 years of L-serine treatment (~600 mg/kg), a patient with a an LoF *GRIN2B* variant displayed improvement in behavioral and cognitive symptoms (Krey et al., 2022). In contrast, L-serine treatment (~500 mg/kg) led to a rapid behavioral decline in a patient with a GoF *GRIN2B* variant after only 2 days, signifying the importance of patient-tailored therapies in *GRIN*-related disorders (Krey et al., 2022). Although the above observations are limited to small numbers of individuals who in some cases were also taking other medications, these studies support the use of precision medicine in treating *GRIN*-related disorders. Thus, future treatment options for individuals with *GRIN*-related disorders should take into consideration the consequence a variant imposes on NMDAR function.

## 4 Discussion

Together, the existing data are beginning to provide insight into the pathophysiological mechanisms that underlie *GRIN2B*-related neurodevelopmental disorder and link specific variants to the development of clinical symptoms. The observations made so far underscore the complexity of NMDAR biology and emphasize the importance of comparing the effects of multiple disease-associated variants when evaluating potential cellular mechanisms of the disease. As such, the field is not yet poised to generalize observations made with individual variants to broad classes of mutants. However, it is likely that useful classifications will be possible in the future as more information is gathered regarding the consequences of a variety of variants at each step of neuron and synapse development. Studying individual variants will help the field move toward identifying core neurodevelopmental phenotypes that characterize the disease and lead to its symptoms. In addition, defining common cellular phenotypes and pathophysiological mechanisms will be informative with regards to which potential therapeutic approaches might be beneficial for individual patients.

There are several points that are important to consider in future studies of GluN2B-related neurodevelopmental disorder variants:

In an analysis of pathophysiological mechanisms of *GRIN2B*-related neurodevelopmental disorder, it is important to consider the variability in clinical phenotypes that are observed in individuals with the disease. It is not clear what causes this variability in symptoms and co-morbidities. One possibility is that the variability derives from the nature of the mutation (*i.e.*, domain of GluN2B that is altered, the severity of the amino acid change, effects of variant on protein expression, localization and function, etc…). Another possibility is that clinical variability stems from the genetic background of individuals bearing *GRIN2B* variants. Environmental factors may also contribute to symptom variability. Consistent with this idea, it is well-established that NMDAR expression and function are modulated by experience and various environmental factors, including developmental neurotoxins and some therapeutics (Neal et al., 2011; Wills et al., 2012; Alavian-Ghavanini et al., 2018; Repouskou et al., 2020; Engdahl et al., 2021a; Sheng et al., 2022). In addition, *GRIN2B* is a target for epigenetic control (Rodenas-Ruano et al., 2012; Gulchina et al., 2017; Alavian-Ghavanini et al., 2018; Loureiro et al., 2020; Engdahl et al., 2021a,b; Sheng et al., 2022). It is unclear the extent to which gene × environment interactions determine the symptomatic profile of this disease, but this will be an important avenue for future investigation. Finally, if individual cells preferentially utilize one *GRIN2B* allele over another, variability in disease symptoms could stem in part from differences in usage of the wild-type vs. mutant alleles. To our knowledge, this idea has not yet been explored.

To fully define the mechanisms that link *GRIN2B* variants to clinical phenotypes, it will be essential to compare the effects of GluN2B variants on different types of neurons and in clinically-relevant circuits. The vast majority of studies of *GRIN2B* variants in neurons have used the hippocampus as a model, but other brain areas may be differentially affected by disease-associated variants. In support of this idea, Shin et al. (2020) reported stronger effects of the C456Y variant on mPFC layer 1 neurons when compared to hippocampal CA1 neurons. Similarly, for variants that lead to visual deficits, it makes sense to study the effects of these variants in visual cortical neurons. Furthermore, within the cortex, both glutamatergic and GABAergic neurons express GluN2B in their dendrites; however, the vast majority of studies on GluN2B variants and function have focused on glutamatergic neurons. As another example, it has been proposed that regulation of dendritogenesis by NMDARs is cell-type specific (Lohmann and Wong, 2005; Espinosa et al., 2009), so disease variants may only regulate dendrite morphogenesis in a subset of neurons.

It is also worth noting that different brain areas have unique spatiotemporal GluN2B expression profiles and mature at different ages. For example, since the PFC matures much later than the primary sensory cortex, PFC neurons may be sensitive to disruptions in GluN2B at more advanced ages compared to visual cortical neurons. This also underscores the importance of expressing mutant GluN2B subunits during sensitive periods, most likely when endogenous synthesis of GluN2B normally begins and occurs at the highest rates, when studying neurodevelopmental phenotypes.

While interpreting experiments evaluating disease-associated GluN2B variants in neurons, non-channel functions of NMDARs should be considered. It has become increasingly clear in recent years that NMDARs serve functions that are independent of their ion channel activity (Dore et al., 2017; Montes de Oca Balderas, 2018; Burket and Deutsch, 2019; Keith et al., 2019). Therefore, both ionotropic and unconventional, ion flux-independent functions of NMDARs represent possible cellular mechanisms linking pathogenic *GRIN2B* variants to clinical phenotypes.

Another issue to consider when evaluating the effects of disease-associated variants on neuronal development is whether the expression of truncated GluN2B protein is an appropriate model for *GRIN2B* variants that produce premature stop codons. In most cells, premature termination codons (PTCs) recruit the nonsense mediated decay (NMD) machinery, which then degrades the mRNA to prevent the production of truncated proteins (Kurosaki et al., 2019; Lee et al., 2021). In general, NMD does not target transcripts when the PTC occurs within 50–55 nucleotides of the last exon-exon junction or within the last exon unless a long 3’UTR is generated (Kurosaki et al., 2019; Lee et al., 2021). This raises the possibility that transcripts generated from many *GRIN2B* variants with PTCs would be targeted for degradation, producing haploinsufficiency rather than truncated GluN2B protein. However, NMD appears to be less robust in neurons compared to most cells, likely rendering neurons vulnerable to the production of truncated proteins from PTC-containing transcripts (Lee et al., 2021). Strikingly, NMD is particularly suppressed in neurons during embryonic and postnatal development (Lee et al., 2021), coincident with the period of high GluN2B expression. These observations suggest that truncated GluN2B may be produced during neuron development. Interestingly, downregulation of NMD during development is necessary for neuron differentiation and maturation (Bruno et al., 2011; Lou et al., 2014), stages of neuronal development that are negatively affected by the expression of disease-associated GluN2B variants, as described above. Ultimately, empirical evidence is necessary to determine the extent to which truncated GluN2B is produced from each truncation variant.

A final important issue is the use of *Grin2b* knockout animals to understand *GRIN2B*-related neurodevelopmental disorder pathophysiology. It is common practice in the study of single-gene disorders to use knockout mice to model disease pathogenesis. While knock-out animals are an invaluable tool, they are not without caveats for the study of disease pathophysiology. Given that individuals that harbor *de novo* *GRIN2B* variants have one wild-type allele and one mutant allele, heterozygous knockout animals provide high face validity for any variants that result in haploinsufficiency. However, it is worth noting that loss of expression from one allele may not automatically halve the GluN2B-containing NMDARs at the postsynaptic plasma membrane. For example, the elimination of one *Grin2b* allele in heterozygous knockout neurons did not reduce GluN2B-dependent post-synaptic responses (Espinosa et al., 2009) or resulted in only partial haploinsufficiency with regard to NMDAR-EPSC properties (Elmasri et al., 2022). This may be a result of cellular mechanisms that control the amount of GluN2B-containing NMDARs at synapses. For example, posttranslational modifications and protein interactions dynamically regulate the trafficking of NMDARs through the biosynthetic pathway, surface delivery, and mobility between subcellular compartments such as synaptic vs. extrasynaptic domains or between the plasma membrane and recycling endosomes (Sanz-Clemente et al., 2013; Horak et al., 2014; Gardoni and Di Luca, 2021). Furthermore, the ultimate effects of heterozygous knockout on levels of GluN2B-containing NMDARs at synapses may vary with brain area, neuron type, and age. This is illustrated by the observation that in visual cortical neurons, expression of GluN2B at synapses is unaffected by transgenic over-expression of GluN2B mRNA (Philpot et al., 2001), while over-expression of GluN2B increased synaptic NMDARs in hippocampal and striatal neurons (Tang et al., 1999; Duan et al., 2018). Therefore, it is essential to empirically determine whether heterozygous *Grin2b* knockout affects the number of GluN2B-containing NMDARs at synapses for each neuron subtype, synapse type, and age studied.

Heterozygous *Grin2b* knockout models may be insufficient to model variants that produce GluN2B subunits that are capable of co-assembly with wild-type subunits or competition for interactions with GluN2B-binding partners. As mentioned above, if mutant subunits co-assemble with wild-type subunits, mutant GluN2B may act in a dominant-negative manner rather than reflecting simple haploinsufficiency. Consistent with this idea, several studies highlighted above have reported evidence for dominant-negative effects of various GluN2B variants (Li et al., 2019; Sceniak et al., 2019; Bahry et al., 2021; Kellner et al., 2021). Comparisons between the expression of mutant GluN2B (in the presence of wild-type GluN2) and heterozygous knockout of *Grin2b* will be useful for determining the extent to which cellular phenotypes best reflect haploinsufficiency or dominant-negative effects.

## Author Contributions

All authors contributed to the writing and editing of the manuscript. All authors contributed to the article and approved the submitted version.

## Footnotes

^1^ https://www.ncbi.nlm.nih.gov/clinvar/?gr=0

^2^ https://alf06.uab.es/grindb/home

^3^ www.curegrin.org

^4^ http://GRIN2B.com

^5^ https://grin-portal.broadinstitute.org

^6^ https://www.simonssearchlight.org

^7^ https://webapp2.pharm.emory.edu/cferv/
